# Supplementary material for: Haploinsufficiency of autism spectrum disorder candidate gene NUAK1 impairs cortical development and behavior in mice
Source: Nat Commun. 2018 Oct 16;9:4289. doi: 10.1038/s41467-018-06584-5 (PMC6191442; doi:10.1038/s41467-018-06584-5)
Supplement: Supplementary file 1 — Supplementary Information [file 41467_2018_6584_MOESM2_ESM.pdf]

## SUPPLEMENTARY INFORMATION

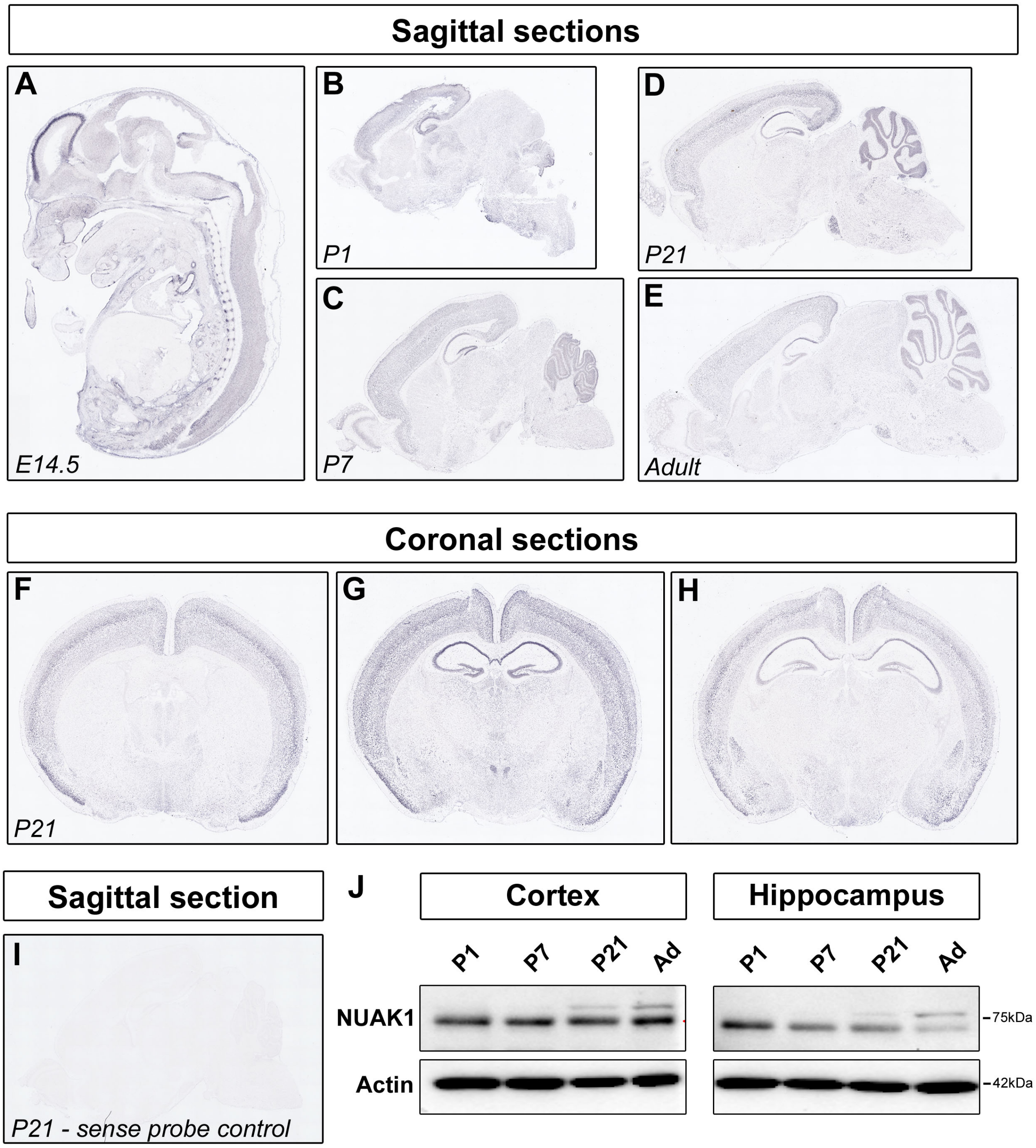


**Supplementary Figure 1: Expression of the NUAK1 gene in the mouse brain**

(A-E) Expression of *Nuak1* in mouse embryo (A) or in sagittal sections of the brain at the indicated ages (Adult: >90 days old) (B-E).

(F-H) Coronal sections of mouse brain at P21 at various cut planes.

(I) Sagittal section at P21 hybridized with a NUAK1 sense probe (negative control)

(J) Western-blot analysis of NUAK1 expression in the cortex or hippocampus of wild-type mice at the indicated ages (Ad: Adult).

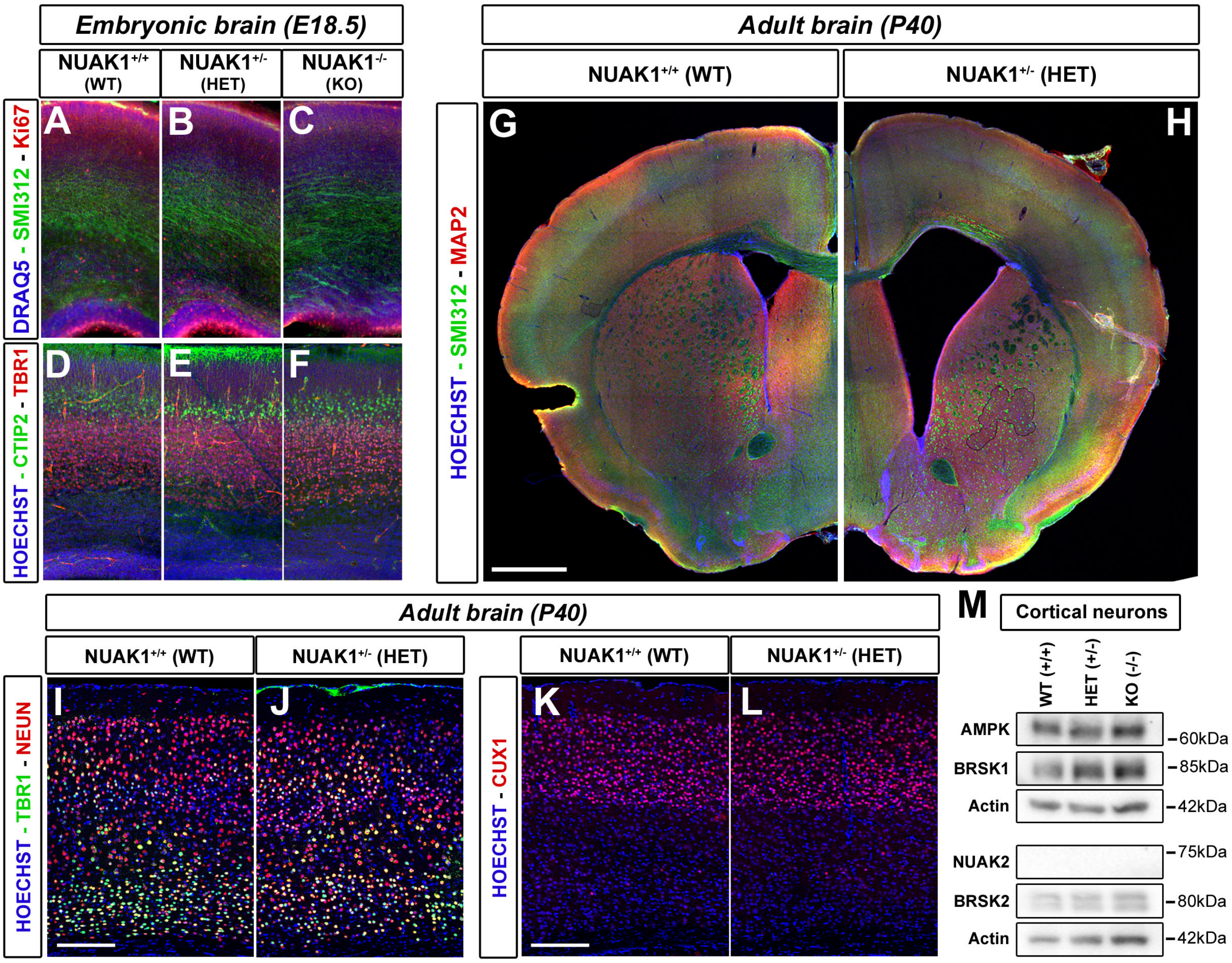


**Supplementary Figure 2: Histochemistry in the brain of WT, HET and KO embryos**

(A-F) Detail of the cortex from coronal sections of E18.5 mouse brains of the indicated genotypes. (A-C) Staining with the axonal marker SMI312 and the proliferation marker Ki67. (D-F) Staining with the layer 5 marker CTIP2 and the layer 6 marker TBR1.

(G-H) Coronal sections of P40 brains of WT and HET mice stained with markers for axons (SMI312, green) or somatodendritic compartments (MAP2, red). Scale bar 1mm.

(I-L) Detail of the cortex from coronal sections of P40 brains of WT and HET mice. (I-J) Staining with the deep layer marker TBR1 and the pan-neuronal marker NeuN. (K-L) Staining with the layer 2-4 marker CUX1. Scale bar 250 µm.

(M) Western-blot analysis in 5DIV cortical neurons of WT, HET and KO animals with the indicated antibodies.

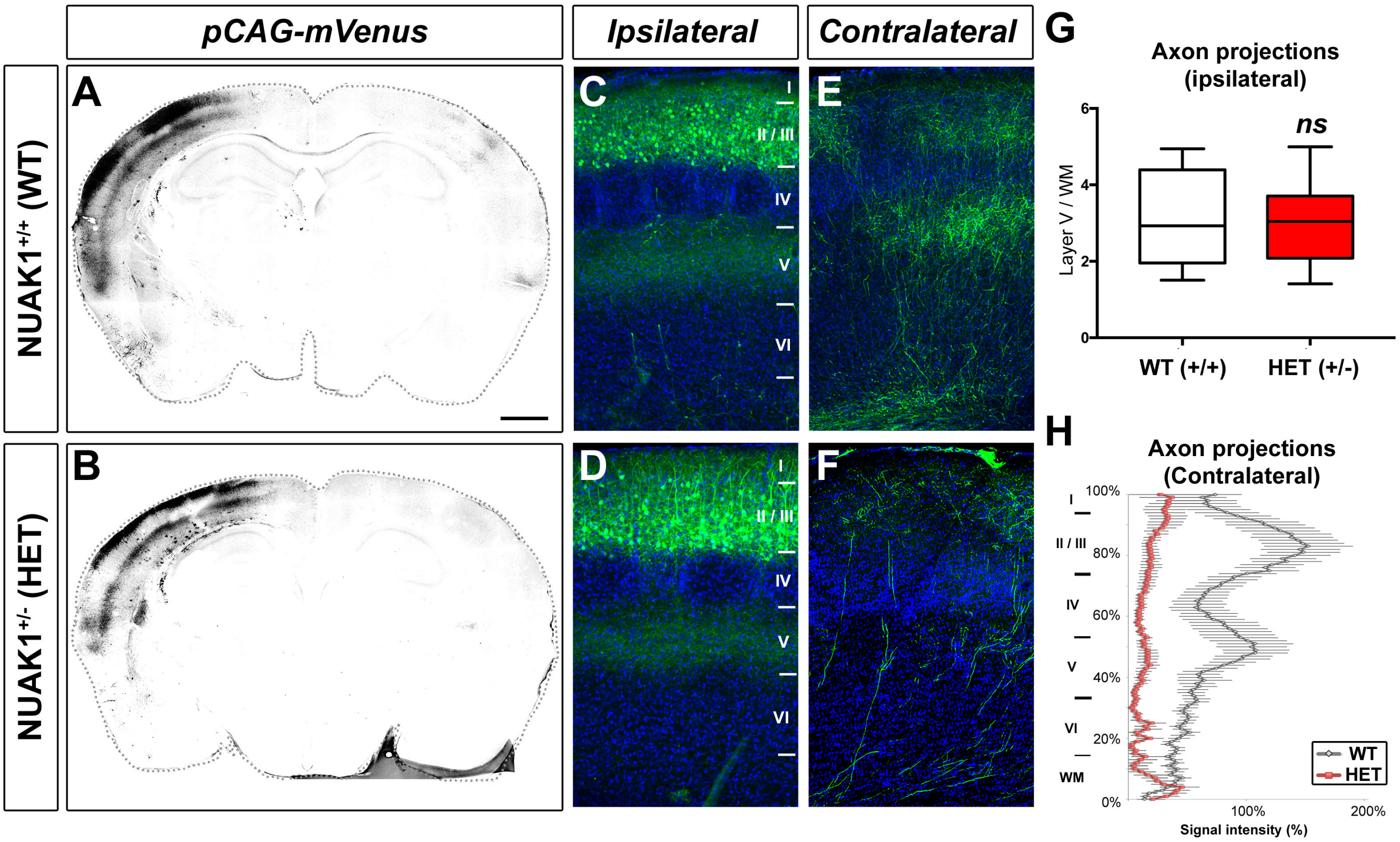


**Supplementary Figure 3: Branching defect in are persistent in older NUAK1 HET mice**

(A-B) Representative coronal sections of brains of WT and HET mice at age P90 upon electroporation of a plasmid encoding the fluorescent protein mVenus. Scale bar 1mm.

(C-F) Histochemistry of the ipsilateral (C-D) or contralateral (E-F) side of $NUAK1^{+/+}$ and $NUAK1^{+/-}$ mice at P90 following in utero electroporation with mVenus. Blue: Hoescht.

(G-H) Quantification of normalized mVenus fluorescence in Layer 5 of the ipsilateral cortex (median, $25^{th}$ and $75^{th}$ percentile) (G) and along a radial axis in the contralateral cortex (Average ± SEM) (H). $N_{WT}$=10, $N_{HET}$=8. Analysis: Two-tailed unpaired T-test (G). ns: p>0.05.

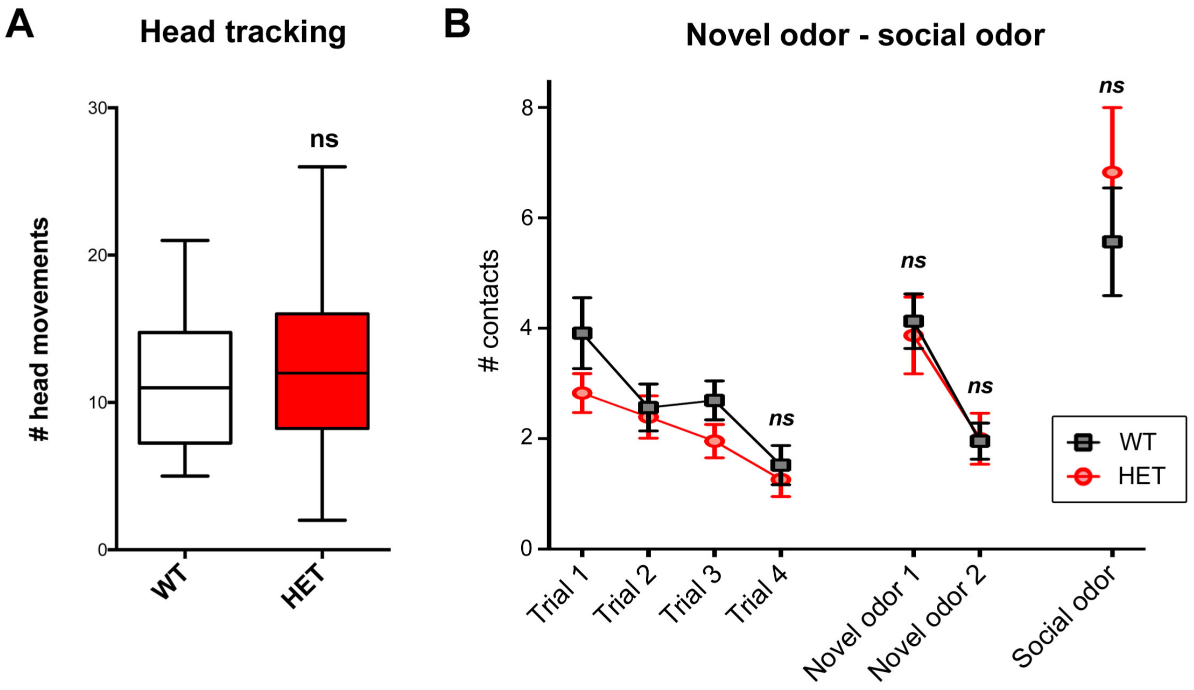


**Supplementary Figure 4: Visual function and response to novel and social odor of NUAK1 HET mice**

(A) Head tracking assay. Quantification of head movements following a visual cue moving forward or backward alternatively. There was no significant effect of genotype ($t(46) = 0.54$, $p = 0.59$). $N_{WT}=24$, $N_{HET}=24$. Analysis: Unpaired T-test. ns: $p>0.05$.

(B) Novel and social odor assay. Number of contacts was quantified in repeated trial sessions with WT and NUAK1$^{+/-}$ mice. There was no effect of genotype ($F(1,308) = 0.32$, $p = 0.57$). $N_{WT}=24$, $N_{HET}=24$. WT and NUAK1$^{+/-}$ groups were compared by 2-way ANOVA followed by Bonferroni's multiple comparison. ns: $p>0.05$.

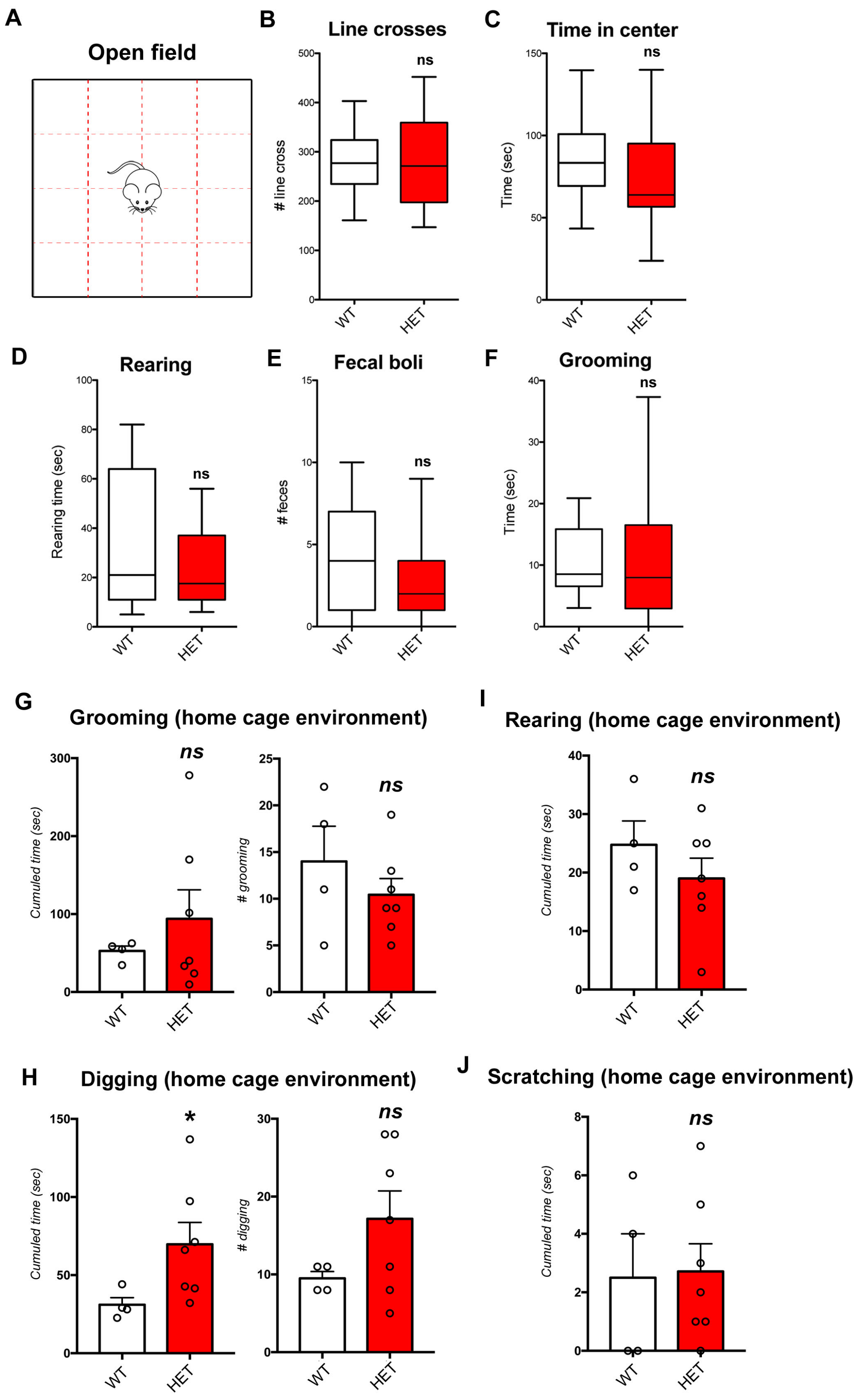


**Supplementary Figure 5: Spontaneous activity of NUAK1 HET mice through the open field assay and in an home cage environment**

(A) Open field assay design.

(B-C) Quantification of line crosses ($t(37) = 0.38$, $p = 0.70$; D) and time spent in center ($t(37) = 0.89$, $p = 0.38$; E) did not reveal any significant change in spontaneous locomotor activity in $NUAK1^{+/-}$ mice.

(D-F) Quantification of rearing ($U = 155$, $p = 0.33$; D), fecal boli ($U = 152.5$, $p = 0.29$; E) and time spent grooming ($U = 164$, $p = 0.47$; F) did not reveal signs of repetitive behavior in $NUAK1^{+/-}$ mice.

Data represents median, $25^{th}$ and $75^{th}$ percentile. $N_{WT}=19$, $N_{HET}=20$. Analysis: Unpaired T-test (B, C) or Mann-Whitney (D, E, F). ns: $p>0.05$.

(G-J) Observation of spontaneous behavior of NUAK1 HET mice in a home cage environment. Spontaneous grooming (G; total time $U = 13$, $p = 0.92$, # events $U = 10$, $p = 0,49$), digging (H; total time $U = 3$, $p = 0.042$, # events $U = 8$, $p = 0,31$), rearing (G; # events $U = 9$, $p = 0,41$) and scratching (G; # events $U = 12$, $p = 0,74$) were quantified over a 10 minutes period. Average ± SEM. $N_{WT}=4$, $N_{HET}=7$. Analysis: Mann-Whitney. ns: $p>0.05$, *: $p<0.05$.

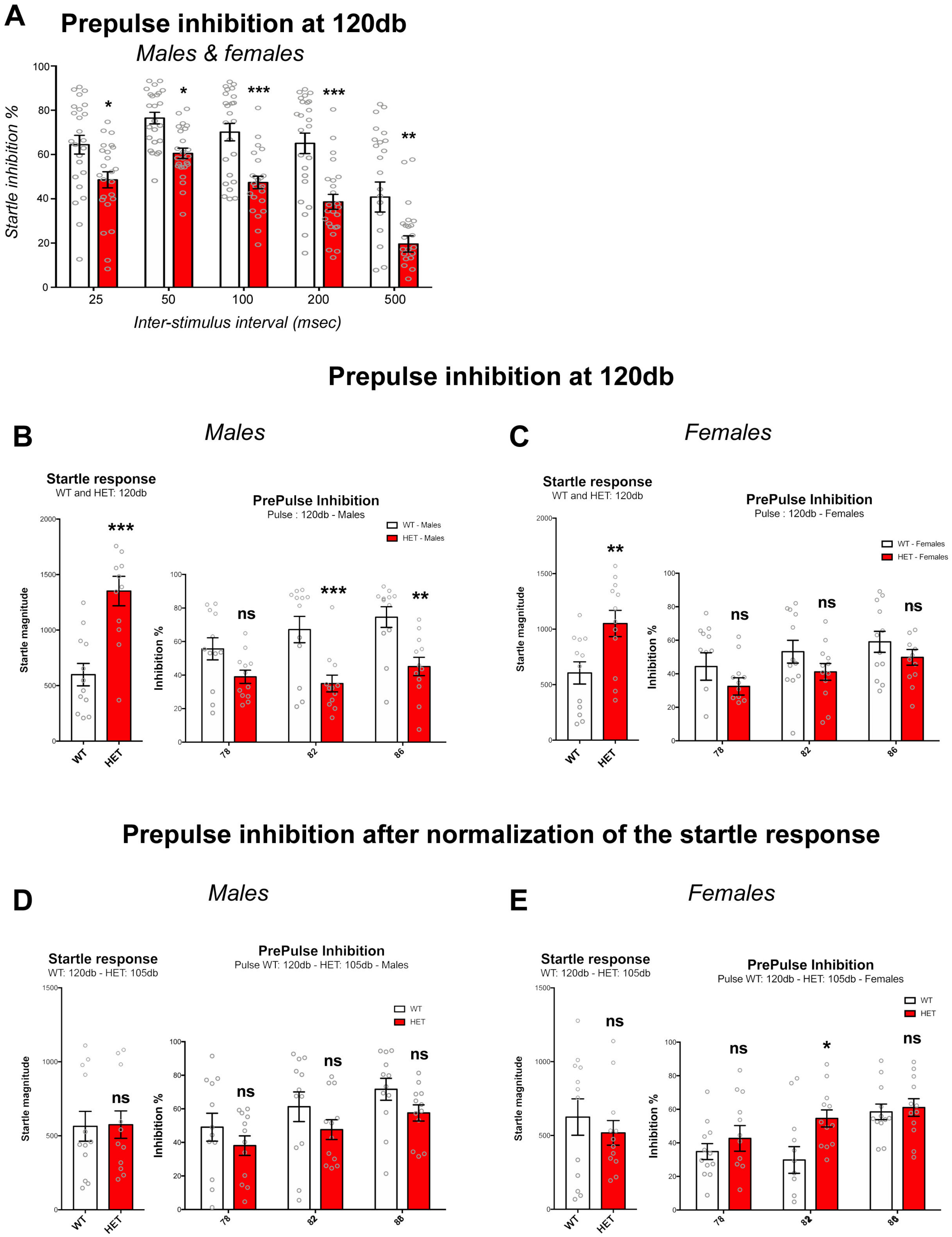


**Supplementary Figure 6: Sex-specific differences in prepulse inhibition response in NUAK1 HET mice**

(A) Effect of the inter-stimulus interval on prepulse inhibition. Pulse 120db, mixed males and females. Average ± SEM. $N_{WT}$=24, $N_{HET}$=24. Analysis: 2-way ANOVA with Bonferroni's multiple comparisons. *: p<0.05, **: p<0.01, ***: p<0.001.

(B-C) Prepulse inhibition (PPI) in WT and NUAK1$^{+/-}$ mice mice. There was a significant difference between male WT and NUAK1$^{+/-}$ mice in PPI when the same startle stimulus was used for each genotype (F(1,22) = 11.6, p = 0.003) as well as a significant interaction between genotype and prepulse intensity (F(2,44) = 3.75, p = 0.03). Post-hoc analyses showed a genotype difference at 82dB (p = 0.0009) and 86 dB (p = 0.0025). There was no difference between WT and NUAK1$^{+/-}$ females using a 120dB startle intensity.

(D-E) Prepulse inhibition assay following startle response normalization in mice from (B-C). HET mice were subject to a milder tone (105db) to compensate for increased startle response. When startle was normalized for PPI analyses, the genotypic difference in males was no longer significant, although a trend was there. In females, there was a significant interaction between genotype and prepulse intensity (F(2,44) = 5.55, p = 0.007) with NUAK1$^{+/-}$ mice showing a greater inhibition at 82dB (p < 0.05; albeit the WT mice appear to the unusual group in this case).

Quantification (B-E): Average ± SEM. All groups of 12 animals. Analysis for Startle response by unpaired T-test. Analysis for PPI by 2-way ANOVA with Bonferroni's multiple comparisons. ns: p>0.05, *: p<0.05, **: p<0.01, ***: p<0.001.

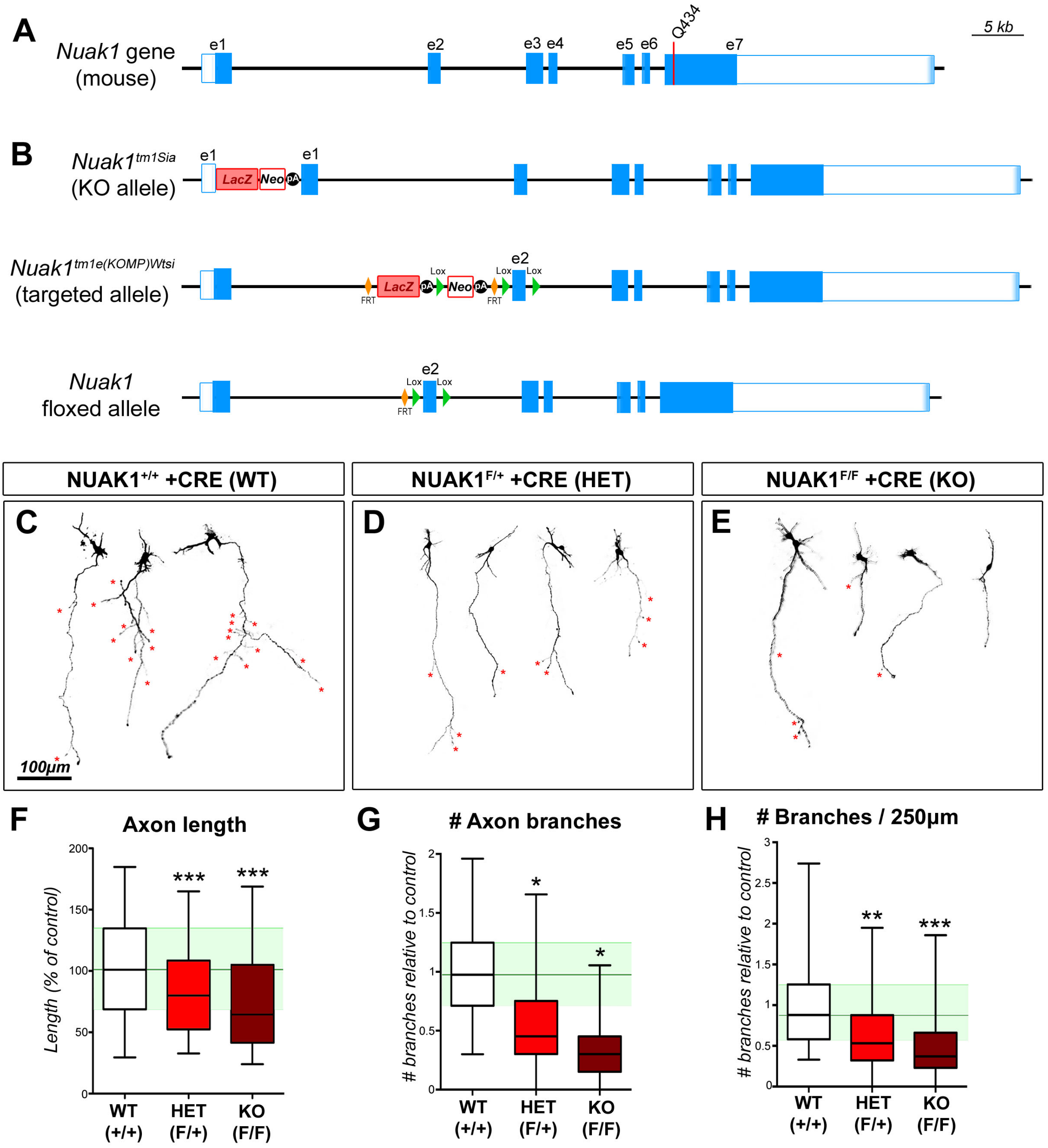


**Supplementary Figure 7: Constitutive (KO) and conditional (floxed) alleles for *Nuak1* in the mouse**

(A) Structure of the *Nuak1* gene in the mouse. Boxes represent the seven exons (e) of the gene. Filled boxes mark the coding sequence. Position of Q434 codon as indicated.

(B) Constitutive (KO) Nuak1 allele as described in Hirano *et al.* [1]. LacZ/Neo cassette was inserted in Exon 1 followed by a PolyA transcription termination sequence (pA).

Targeted allele generated through the KOMP repository. LacZ and Neo genes were flanked by FRT sites. Crossing with FRT-expressing mice was used to generate a conditional (floxed) allele.

(C-E) Representative neurons imaged after 5DIV following ex vivo electroporation of CRE and dissociation of neurons from NUAK1$^{+/+}$ (WT), NUAK1$^{F/+}$ (HET) and NUAK1$^{F/F}$ (KO) animals. Neuron morphology was visualized through mVenus expression.

(F-H) Quantification of axon length (F), number of branches per axon (G) and normalized branch number (H). Data represents median, 25$^{th}$ and 75$^{th}$ percentile (F) or Average ± SEM (G-H). $N_{WT}$=22, $N_{HET}$=113, $N_{KO}$=80. Analysis: Kruskal-Wallis test with Dunn's multiple comparison. *: p<0.05, **: p<0.01, ***: p<0.001.

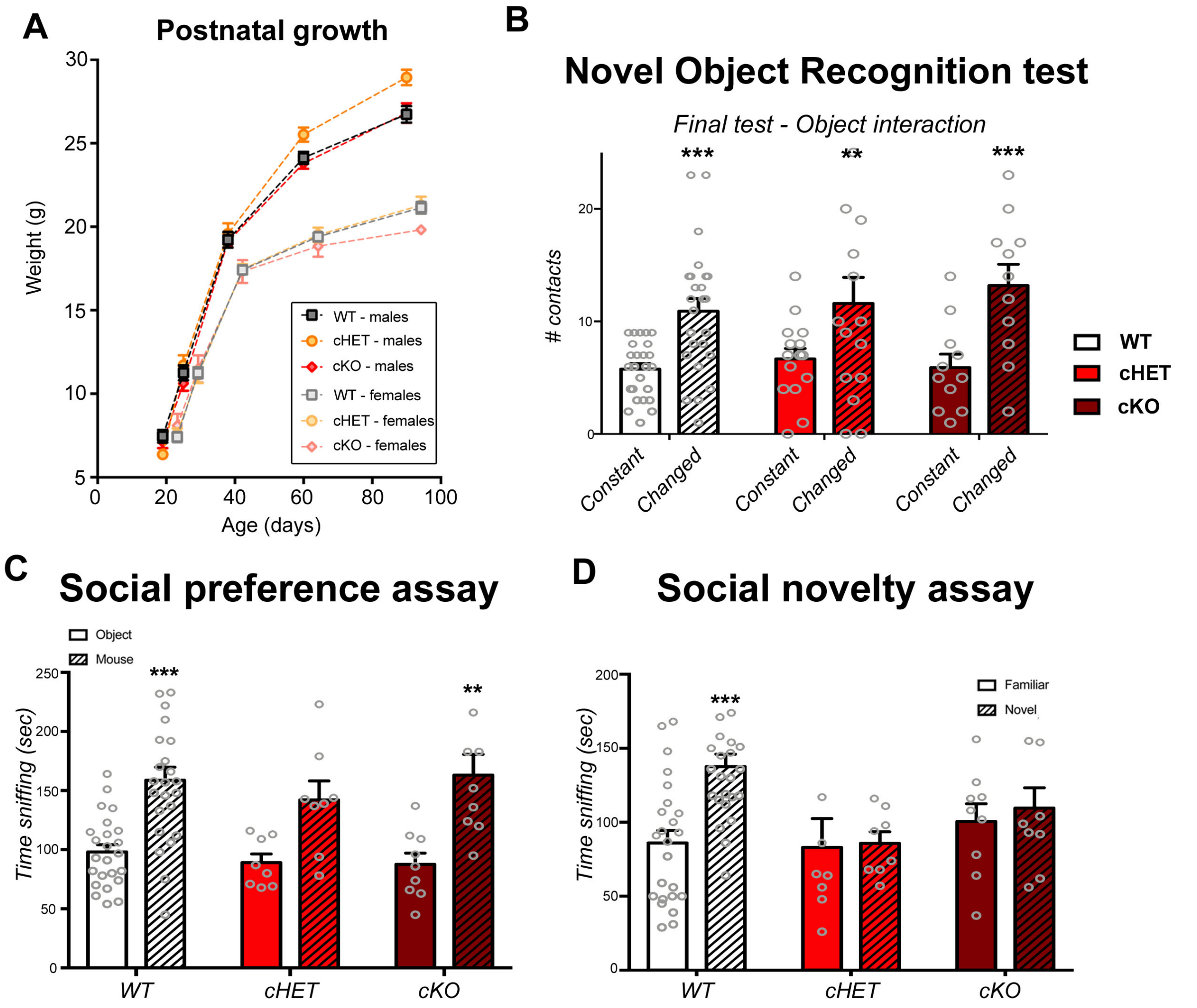


**Supplementary Figure 8: Constitutive (KO) and conditional (floxed) alleles for *Nuak1* in the mouse**

(A) Postnatal growth curve of male and female mice upon inactivation of NUAK1 in the dorsal telencephalon.

(B) Quantification of the number of contacts during the novel object recognition assay did not reveal any genotype-associated difference in object recognition. $N_{WT}$=25, $N_{cHET}$=15, $N_{cKO}$=11. Analysis: 2-way ANOVA with Bonferroni's multiple comparisons.

(C-D) Social preference assay (C) and preference for social novelty (D) in NUAK1 cKO mice measured through direct interaction time. Data: Average ± SEM. $N_{WT}$=24, $N_{cHET}$=8, $N_{cKO}$=9. Analysis: 2-way ANOVA with Bonferroni's multiple comparisons. ns: $p>0.05$, **: $p<0.01$, ***: $p<0.001$.

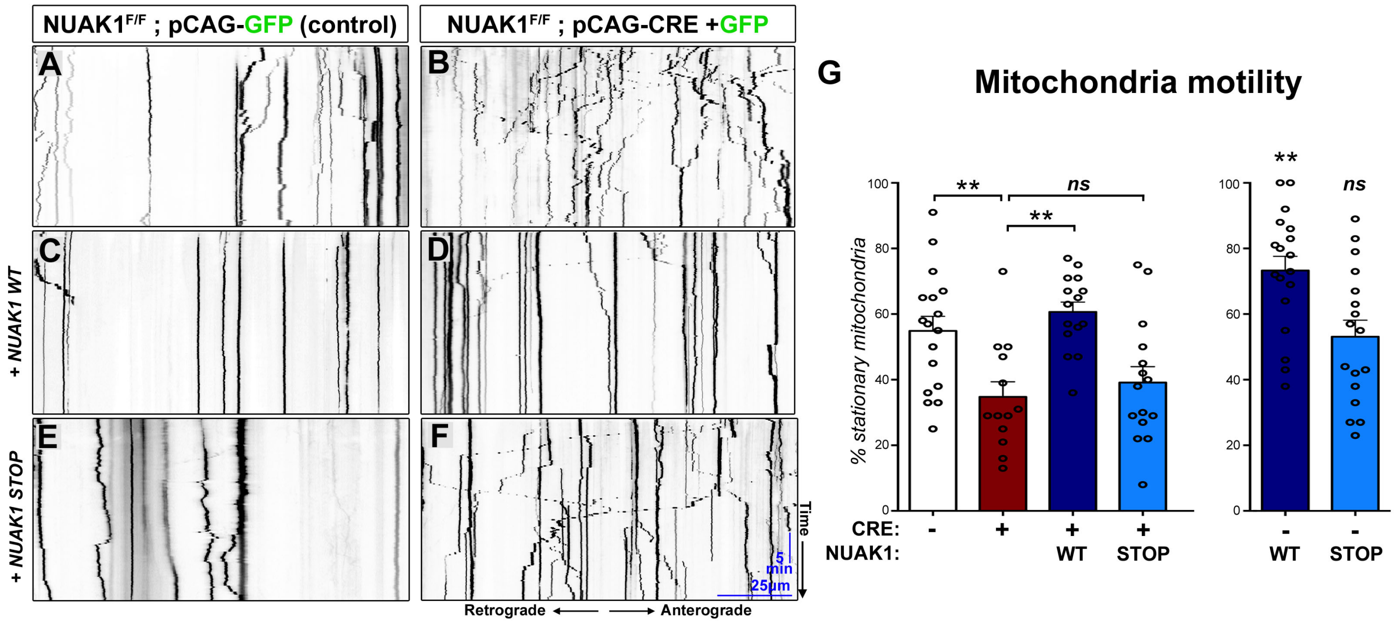


**Supplementary Figure 9: NUAK1 mutant protein fails to rescue mitochondria arrest**

(A-F) Representative kymographs of axons of NUAK1$^{F/F}$ neurons at 5DIV and electroporated with pCAG-GFP control vector (A, C, E) or pCAG-CRE vector (B, D, F) together with wild-type NUAK1 (C, D) or NUAK1 STOP mutant (E, F). Mitochondria were visualized through expression of mito-DsRed. Blue bar (vertical): 5 min. Blue bar (horizontal): 25 µm.

(G) Quantification of the percentage of stationary mitochondria in the axon over 30 minutes. Average ± SEM. $N_{CTL}$=17, $N_{CRE}$=13, $N_{CRE+WT}$=15, $N_{CRE+STOP}$=15, $N_{WT}$=18, $N_{STOP}$=17. Data from 3 independent cultures. Analysis: Student's T-test. ns: p>0.05, **: p<0.01.

**Raw data (blots) from Figure 1A**

**10% SDS-PAGE - Protein Ladder Dual Color (BioRad)**

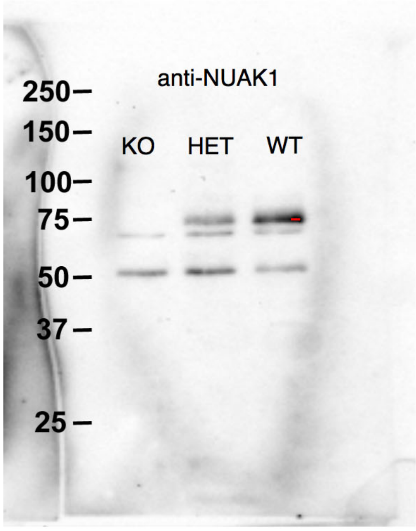


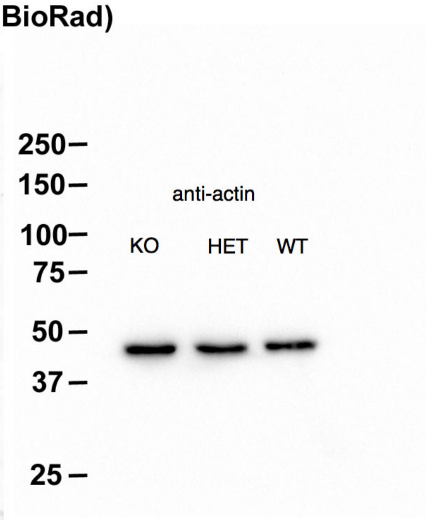


**Raw data (blots) from Supplementary figure 1J**

**10% SDS-PAGE - Protein Ladder Dual Color (BioRad)**

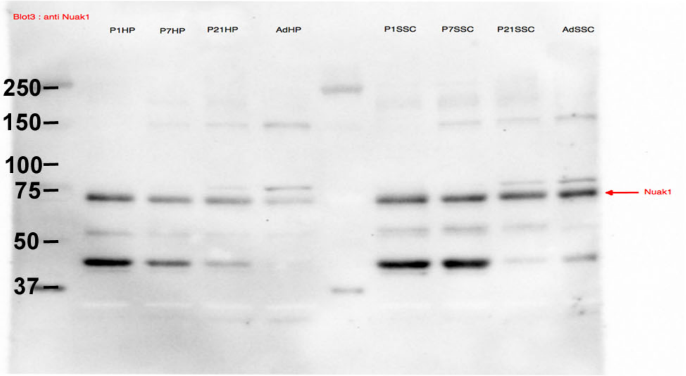


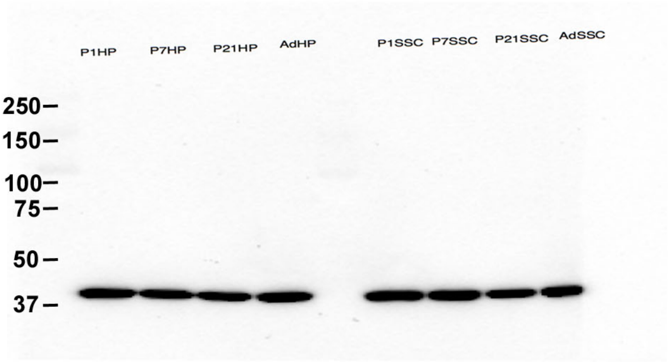


**Supplementary Figure 10: Raw data (blots) used in Figure 1A and Supplementary Figure 1J**

The blots used for making Figure 1A and Supplementary Figure 1J are included alongside with annotations and molecular weight.

| Study | Gene name | Location (human) | Variant | Type | Location (mouse) | Comment |
|---|---|---|---|---|---|---|
| Iossifov et al., 2012 [2] | *Stk11* | 19:1223103 | sub(C->T) | missense | N/A | Position doesn't exist in mouse |
| O'Roak et al., 2012 [3] | *Brsk2* | 11:1466812 | del(3) | in frame | 7:141988126 | Deletion in UBA domain |
| Iossifov et al., 2014 [4] | *Mark2* | 11:63665704 | sub(C->T) | synonymous | 19:7287378 | Intron 3 - Exon 4 junction |
| Iossifov et al., 2014 [4] | *Mark3* | 14:103969422 | sub(C->T) | missense | 12:111655473 | Mutation in KA1 domain (phospholipid binding) |
| Iossifov et al., 2014 [4] | *Nuak1* | 12:106460608 | sub(G->A) | missense | 10:84374274 | A to V substitution in C-term region |
| Iossifov et al., 2012 [2] | *Nuak1* | 12:106461269 | sub(G->A) | nonsense | 10:84374923 | Generates PTC in last exon |
| Iossifov et al., 2014 [4] | *Prkaa1* | 5:40775592 | ins(TGA) | In frame | 15:5164723 | Mutation in kinase domain |
| Iossifov et al., 2014 [4] | *Sik1* | 21:44839867 | sub(A->T) | missense | 17:31849656 | Mutation in UBA domain |
| Iossifov et al., 2014 [4] | *Sik1* | 21:44840121 | sub(G->A) | missense | 17:31849935 | Mutation in UBA domain. CAMK1 phosphorylation site. |
| Iossifov et al., 2014 [4] | *Sik3* | 11:116732054 | sub(G->A) | synonymous | 9:46209600 | |
| Iossifov et al., 2014 [4] | *Sik3* | 11:116766982 | ins(AAAT) | frame-shift | 9:46178535 | Mutation in kinase domain |
| Iossifov et al., 2014 [4] | *SNRK* | 3:43389739 | sub(A->G) | missense | 9:122167150 | |

**Supplementary Table 1: genetic mutations identified in LKB1 and AMPK-RK genes**

Mutations were identified in the indicated studies. Location refer to the human GRCh37/hg19 and mouse GRCm38/mm10 assembly.

## SUPPLEMENTARY METHODS

### Reagents, chemicals and antibodies

List of primary antibodies and fluorescent dyes are indicated in the table below

| ANTIBODY | MANUFACTURER | DILUTION |
|---|---|---|
| ***Primary antibodies*** | | |
| Chicken anti-GFP | Aves Laboratories<br>Cat GFP-1020 | 1:2,000 (IHC-ICC) |
| Mouse Pan axonal Neurofilament marker (SMI312) | Covance<br>Cat SMI-312R | 1:2,000 (IHC-ICC) |
| Rabbit anti-MAP2 | Millipore<br>Cat AB5622 | 1:2,000 (IHC) |
| Rabbit anti-ARK5 (NUAK1) | Cell signaling<br>Cat #4458S | 1:1,000 (WB) |
| Rabbit anti-SNARK (NUAK2) | Cell signaling<br>Cat #4100S | 1:1,000 (WB) |
| Rabbit anti-BRSK1 (D10F2) | Cell signaling<br>Cat #5934S | 1:1,000 (WB) |
| Rabbit anti-BRSK2 (D29B6) | Cell signaling<br>Cat #5460S | 1:1,000 (WB) |
| Rabbit anti-AMPK | Cell signaling<br>Cat #5831S | 1:1,000 (WB) |
| Mouse anti-Actin, clone C4 | MP biomedicals<br>Cat 08691001 | 1:10,000 (WB) |
| Rabbit anti-TBR1 | Abcam<br>Cat ab31940 | 1:500 (IHC) |
| Rabbit anti-CUX1 (CDP) | Santa Cruz<br>Cat sc-13024 | 1:500 (IHC) |
| Rat anti-CTIP2, clone 25B6 | Abcam<br>Cat ab18465 | 1:500 (IHC) |
| Mouse anti-NeuN, clone 1B7 | Abcam<br>Cat ab104224 | 1:2,000 (IHC) |
| Mouse anti-Ki67, clone B126.1 | Abcam<br>Cat ab8191 | 1:1,000 (IHC) |
| Mouse anti-Tubulin beta 3 (TUBB3) (Tuj1) | Covance<br>Cat MMS-435P | 1:2,000 (IHC) |
| Mouse anti-alpha-tubulin, clone DM1A | Millipore<br>Cat 05-829 | 1:2,000 (WB) |
| ***Secondary antibodies and dyes*** | | |
| DRAQ5 Fluorescent Probe Solution (5 mM) | Thermo scientific<br>Cat 62251 | 1:5,000 (IHC) |
| Hoechst 33258, Pentahydrate, 10mg/mL in water | Pierce<br>Cat H3569 | 1:5,000 (IHC) |
| Goat anti-Chicken, Alexa488 | Invitrogen<br>Cat A11-039 | 1:2,000 (IHC-ICC) |
| Donkey anti-Rabbit, Alexa546 | Invitrogen<br>Cat A10-040 | 1:2,000 (IHC-ICC) |
| Donkey anti-Mouse, Alexa647 | Invitrogen<br>Cat A31-571 | 1:2,000 (IHC-ICC) |

WB, Western blot; ICC, immunocytochemistry; IHC, immunohistochemistry.

**Behavioral assay**

*Description of cohorts and order of experiments.*

For NUAK1$^{+/-}$ mice, age-related cohorts of 24 mice (12 males and 12 females) for each genotype were used for all behavioral assays with the exception of the Novel object assay, Fear Conditioning and Home-Cage observations (see below). In order to minimize the overall number of animals used in the study, mice were subjected to several tests in the order indicated below with a 5-10 days pause separating each test:

(1) Open field test

(2) Optomotor test of vision

(3) Non-social and social odor olfactory test

(4) Barnes maze test

(5) Social interaction test

(6) Startle/prepulse inhibition (PPI) of sensory gating

The novel object assay was performed on two cohorts (first cohort of 8 mice and second cohort of 12 mice) for NUAK1$^{+/+}$ and NUAK1$^{+/-}$ mice. The Fear Conditioning experiment was performed in age related NUAK1$^{+/+}$ and NUAK1$^{+/-}$ littermate female mice. After a resting period of 7 days, home-cage observations of behavior was performed on the same mice.

For NUAK1 cHET and cKO mice, the novel object was performed first, followed by the social interaction test 7 days later. The tests were performed on age-matched animals from 6 independent litters (10-12 weeks old). Fear Conditioning was performed in 12 week old female NUAK1 cKO mice and wild type littermates.

*Optomotor test.* The optomotor test allows for assessment of visual ability and consists of a stationary elevated platform surrounded by a drum with black and white striped walls. Each mouse was placed on the platform to habituate for 1 minute and then the drum rotated at 2rpm in one direction for 1 minute, was stopped for 30 sec, and then rotated in the other direction for 1 minute[5]. The number of head tracks (15 degree movements at speed of drum) was recorded. Blind mice do not track the moving stripes.

*Dishabituation to non-social and social odors olfactory test*. The assay is based on the decreased sniffing of a novel odor upon habituation and increased sniffing upon dishabituation when the odor is changed [6-8]. A cotton swab containing either an anise or a banana odorant was presented to mice placed in clean cages for 4 trials of 2 min each, separated by 1 min periods and numbers of active sniffing episodes were measured. The novel odor test consisted of switching to the odorant not used in the initial trials and continuing with 2 trials. Finally, the effects of subsequent introduction of a cotton swab saturated with a social odor obtained by swiping the swab across the bottom of a cage of novel C57BL/6J mice was assessed, again in two 2-min trials.

*Open field test.* Mice were placed in a square white Plexiglas (50 x 50 cm) open field that is divided into 16 squares (12 outer and 4 inner) of equal areas. The walls of the open field are 22 cm high. Each animal was placed in the center of the field and several behavioral parameters (time spent in the inner squares, number of squares crossed, number of fecal boli, rearings and time spent grooming) were recorded during a 10-minute observation period.

*Novel object test.*

Mice were individually habituated to a 51cm x 51cm x 39cm open field for 5 min. Mice were then tested individually with two identical objects (objects ‘$A_1$’ and ‘$A_2$’) placed in the field (either two 250 ml amber bottles or two clear plastic cylinders 6x6x16cm half filled with glass marbles). Object and side of the cage was randomly assigned. Each mouse was allowed to explore for 5 min with the objects present. After two such trials (each separated by 1 minute in a holding cage), the mouse was tested in the object novelty recognition test in which a novel object (object ‘B’; for example, an amber bottle if the cylinders were initially used) replaced one of the familiar objects. All objects and the arena were thoroughly cleaned with 70% ethanol between trials to remove odors. Behavior was video recorded and then scored for contacts (touching with nose or nose pointing at object and within 0.5 cm of object). Habituation to the objects across the familiarization trials (decreased contacts) was an initial measure of learning and then renewed interest (increased contacts) in the new object indicated successful object memory.

*Barnes maze test.* The maze used was an opaque Plexiglas disc 75 cm in diameter elevated 58 cm above the floor by a tripod. Twenty holes, 5 cm in diameter, were located 5 cm from the perimeter, and a black Plexiglas escape box (19 x 8 x 7 cm) was placed under one of the holes. Distinct spatial cues were located all around the maze and were kept constant throughout the study.

On the first day of testing, a training session was performed, which consisted of placing the mouse in the escape box and leaving it there for one minute. One minute later, the first session was started. At the beginning of each session, the mouse was placed in the middle of the maze in a 10 cm high cone-shaped silver start chamber.

After 10 seconds the start chamber was removed, a buzzer (80 dB) and a light (400 lux) were turned on, and the mouse was set free to explore the maze. The session ended when the mouse entered the escape tunnel or after 3 min elapsed. When the mouse entered the escape tunnel, the buzzer was turned off and the mouse was allowed to remain in the dark for one minute. The tunnel was always located underneath the same hole (stable within the spatial environment), which was randomly determined for each mouse. Mice were tested once a day for 4 days for the acquisition portion of the study. A probe test was performed on the day following the final acquisition trial, during which the escape tunnel was removed and the mouse was allowed to freely explore the maze for 3 min. The time spent in each quadrant was determined and the percent time spent in the target quadrant (the one originally containing the escape box) was compared with the average percent time in the other three quadrants. Finally the mice were tested again four weeks later to allow for the examination of long-term memory. Each session was videotaped and scored by an experimenter blind to the genotype of the mouse.

*Three chambers social interaction test.* Mice were placed in a three-chambered Plexiglas box, with each chamber measuring 20cm x 40.5 cm x 22 cm. Dividing walls were clear with small semicircular openings (3.5 cm radius) allowing access into each chamber. The middle chamber was empty, and the two outer chambers contained small, round wire cages (Galaxy Cup, Spectrum Diversified Designs, Inc., Streetsboro, OH) during testing. Mice were habituated to the entire apparatus without the wire cages for 5 minutes. To assess social preference, mice were returned to the middle chamber with a stranger mouse (C57BL/6J of the same sex being tested, habituated to the wire cage) in one of the wire cages in an outer compartment and an empty wire

cage in the other outer compartment. Time spent in the chamber with the novel mouse and time spent in the chamber with the novel object was recorded for 5 minutes as was number of entries into each chamber. For the social novelty test, mice were returned to the middle chamber, this time with the original mouse (familiar mouse) in its chamber and a new unfamiliar mouse (novel mouse) in the previously empty chamber. Again, time spent in each chamber and number of chamber transitions was recorded for 5 minutes. Interval between habituation and social preference assay, and between social preference and social novelty assay was 30 seconds. The position of the object, mouse and novel mouse was assigned in a random manner.

*Acoustic startle/pre-pulse inhibition (PPI) test.* Startle is elicited by a brief tone stimulus between 90 and 120 dB. PPI is the reduction in the startle response observed when the startle stimulus is preceded by a quieter sound stimulus. Startle and pre-pulse inhibition testing was performed using San Diego Instruments startle chambers (SR-Lab; San Diego, California). These consist of nonrestrictive Plexiglas cylinders 5 cm in diameter resting on a Plexiglas platform in a ventilated chamber. High frequency speakers mounted 33 cm above the cylinders produce all acoustic stimuli, which are controlled by SR-LAB software. Piezoelectric accelerometers mounted under the cylinders transduce movements of the animal, which are digitized and stored by an interface and computer assembly. Beginning at startling stimulus onset, 65 consecutive 1 ms readings were recorded to obtain the peak amplitude of the animal's startle response. A 60 min test session was used, in which pulse values were 90, 95, 100, 105, 110, 115 and 120 dB; pre-pulse intervals will be 25, 50, 100, 200, and 500 ms; and pre-pulse intensities were 78 dB, 82 dB and 86 dB, on a 70-dB background level. Startle pulses were 40 ms in duration and pre-pulses were 20 ms in duration.

All trial types (pulse alone, no-stimulus trials (background only), and prepulse + pulse trials) were presented several times in a pseudorandom order (Barros et al., 2009).
We found a large genotypic difference in startle, with the NUAK1 HET mice showing a greater response between 105 & 120 dB. This difference can make it difficult to interpret PPI as this is represented as a percent change in response. Therefore we designed an additional program which looked at PPI at both 105 dB and 120 dB and comparisons were made between WT mice at 120 dB and NUAK1 HET mice at 105 dB because the startle response at these levels were more equivalent. In this case a 45 min test session was used, in which pulse values were 105 and 120 dB; the pre-pulse interval was 100 ms; and pre-pulse intensities were 78 dB, 82 dB and 86 dB, on a 70-dB background level.

*Fear Conditioning (FC).* The fear conditioning apparatus chamber consists in an isolated cubicle (55x60x57cm) containing a dual visible/IR light source, a noiseless fan, a internal speaker and a video camera. A contextual cage (17x17x25cm) with electrified floor was connected to a power supply providing the cage flooring with calibrated electric pulses. On day 1, mice were placed in the conditioning chamber for 2min, before being exposed to a one tone-shock pairing. The shock (constant current 0,7 mA) was delivered during the last 2 seconds of the tone (60 dB, 2800 Hz, 30 seconds). Mice were left in the apparatus for an additional period of 30 seconds before being returned to their home cage for 24 hours. On Day 2, mice were returned to the apparatus with the same environment (assessing contextual fear conditioning) for 3min with neither tone nor shock. One hour later, mice were placed in the apparatus with a novel environment (new flooring with different texture, new colour and visual cues) in

which they were exposed to the tone for 2 minutes (assessing cued fear conditioning) after 1 minute of habituation.

Electric shocks, light and sound were controlled by the software using the calibrated touchscreen controller (Ugo Basile Fear Conditioning System, Italy). Experiment was run under IR as pre-setted on the apparatus and visible light (30 lux) put on automatically when recording starts. Noteworthy, visible light was not associated with the electric shock. The detection of freezing was automated based on video analysis (EthoVision XT13, Noldus Netherlands) and defined as the complete lack of motion for a minimum of 2sec. The percentage of activity (defined by the software as the percentage of pixel change on the image from one frame to the other) is used as a measure of activity for the training period (day 1). Both the cumulative freezing duration and percentage of activity are reported, for each day of analysis.

*Home cage observations.* Mice were placed in individual cage of the same model as their home cage (polypropylene, floor dimensions 36x20cm, height 18cm) with 500 mL soiled bedding (from their own cage) and 500 mL of clean bedding (Envigo FS14). Food and water were provided ad libitum during the period of the assay. Each animal was recorded for a 40 minutes period with a camera placed on the side of the cage. The first 30 minutes of the assay were considered a period of acclimatation. The spontaneous behaviors (grooming and digging) were analyzed for the last 10 minutes of the test. Quantification (frequency and duration) was quantified by manual timing by a trained experimenter.

**In Situ Hybridization**

In situ hybridization was carried out by the RNA In Situ Hybridization Core facility at Baylor College of Medicine (Houston, TX)[9]. Whole embryos (stage E14.5) or brains of postnatal day 1, 7, 21 (P1, P7, P21) and adult C57Bl/6 mice were collected and fixed in 4% PFA for 2 hours, then embedded in OCT following successive bath in 10, 20 and 30% sucrose-PBS solutions. The embryos and brains were cut using a cryostat and 25µm thick sagittal sections (all stages) or coronal sections (P21) were collected on slides. In situ hybridization was performed using DIG-labeled Nuak1 antisense and sense probes generated using mouse complementary DNA probe IMAGE 6842317 (GenBank CB522124)[10]. Mosaic images were taken using an automated Leica brightfield microscope[11]. The RNA In Situ Hybridization Core at Baylor College of Medicine is, in part, supported by a Shared Instrumentation grant from the NIH (1S10OD016167).

**Cell culture**

HEK293T cells were obtained from ATCC and cultured at 37 °C in 5% CO2. Cells were grown in Dulbecco's modified Eagle's medium (Gibco) with 4.5 g/liter glucose containing 10% fetal bovine serum, 100 units/ml penicillin, and 0.1 mg/ml streptomycin (Gibco). For transfection experiments, cells were plated at $2.10^6$ cells in 10cm culture dishes 24 h before transfection, and then plasmids were transfected into cells using JetPEI (Polyplus Transfection).

**NUAK1 kinase activity**

NUAK1 transfected HEK293T cells were lysed 48 hours following transfection in ice-cold NP-40 buffer (20mM Tris pH 7.5, 150mM NaCl, 2mM EDTA, 1% NP-40, 1X protease and phosphatase cocktail inhibitors). 500µg of proteins were diluted in 1mL

of NP-40 buffer and immunoprecipitated with 2μL of anti-NUAK1 antibody overnight at 4°C. The subsequent day, 50 μL of protein A/G-agarose (Roche) was added to the lysate for 1 hour at 4°C. Agarose bead were then washed 5 times with 1mL of NP-40 buffer, then once with 1mL of Assay buffer (50mM Tris pH 7.5, 150mM NaCl, 10mM $MgCL_2$, 0.1mM EGTA, 1mM DTT). Agarose beads were incubated with the peptide substrate CHKtide (200μM) (Millipore) in presence of 0.1 mM $\gamma^{32}$P-ATP in 50μL of Assay buffer at 30°C. After 30 minutes, the supernatant was applied onto P81 phosphocellulose paper (Millipore) and washed 5 times in phosphoric acid, and once in acetone. Radioactivity was quantified by Cherenkov counting.

## SUPPLEMENTARY REFERENCES


1. Hirano, M. *et al.* A new serine/threonine protein kinase, Omphk1, essential to ventral body wall formation. *Dev Dyn* **235,** 2229–2237 (2006).
2. Iossifov, I. *et al.* De novo gene disruptions in children on the autistic spectrum. *Neuron* **74,** 285–299 (2012).
3. O'Roak, B. J. *et al.* Sporadic autism exomes reveal a highly interconnected protein network of de novo mutations. *Nature* **485,** 246–250 (2012).
4. Iossifov, I. *et al.* The contribution of de novo coding mutations to autism spectrum disorder. *Nature* **515,** 216–221 (2014).
5. Amador-Arjona, A. *et al.* Primary Cilia Regulate Proliferation of Amplifying Progenitors in Adult Hippocampus: Implications for Learning and Memory. *Journal of Neuroscience* **31,** 9933–9944 (2011).
6. Crawley, J. N. *et al.* Social approach behaviors in oxytocin knockout mice: comparison of two independent lines tested in different laboratory environments. *Neuropeptides* **41,** 145–163 (2007).
7. Ryan, B. C., Young, N. B., Moy, S. S. & Crawley, J. N. Olfactory cues are sufficient to elicit social approach behaviors but not social transmission of food preference in C57BL/6J mice. *Behavioural Brain Research* **193,** 235–242 (2008).
8. Yang, M. & Crawley, J. N. Simple behavioral assessment of mouse olfaction. *Curr Protoc Neurosci* **Chapter 8,** Unit 8.24 (2009).
9. Yaylaoglu, M. B. *et al.* Comprehensive expression atlas of fibroblast growth factors and their receptors generated by a novel robotic in situ hybridization platform. *Dev Dyn* **234,** 371–386 (2005).
10. Courchet, J. *et al.* Terminal Axon Branching Is Regulated by the LKB1-NUAK1 Kinase Pathway via Presynaptic Mitochondrial Capture. *Cell* **153,** 1510–1525 (2013).
11. Carson, J. P., Thaller, C. & Eichele, G. A transcriptome atlas of the mouse brain at cellular resolution. *Current Opinion in Neurobiology* **12,** 562–565 (2002).
